# Supplementary material for: Timp2 loss-of-function mutation and TIMP2 treatment in murine model of NSCLC: modulation of immunosuppression and oncogenic signaling
Source: bioRxiv. 2023 Dec 29:2023.12.29.573636. Preprint. [Version 1] doi: 10.1101/2023.12.29.573636 (PMC10793420; doi:10.1101/2023.12.29.573636)
Supplement: Supplement 4 [file NIHPP2023.12.29.573636V1-supplement-4.pdf]

bioRxiv preprint doi: https://doi.org/10.1101/2023.12.29.573636; this version posted December 29, 2023. The copyright holder for this preprint (which was not certified by peer review) is the author/funder. This article is a US Government work. It is not subject to copyright under 17 USC 105 and is also made available for use under a CC0 license.

Supplementary Materials

Supplementary Methods

Derivation of congenic Timp2 mutant mice (mT2): T*imp2* mutant mice were first developed in a mixed genetic background (C57BL/6j and 129/ReJ) (*21*). We created a new congenic strain of mutant *Timp2* mice (mT2) containing a targeted deletion of exons 2 and 3 of the *Timp-2* locus in a clean C57BL/6j genetic background. Marker-assisted breeding was utilized to generate the

bioRxiv preprint doi: https://doi.org/10.1101/2023.12.29.573636; this version posted December 29, 2023. The copyright holder for this preprint (which was not certified by peer review) is the author/funder. This article is a US Government work. It is not subject to copyright under 17 USC 105 and is also made available for use under a CC0 license.

new strain by backcrossing original male mT2 mice (C57BL/6j and 129/ReJ) with wt C57BL/6j females purchased from The Jackson Laboratory. Ten male carriers of the *Timp2* mutation were selected after each backcross and subjected to full genome scans using 96 polymorphic microsatellite markers spanning all autosomal chromosomes. Mice with the highest number of homozygous C57BL/6j markers were selected as breeders for the next generation. mT2 mice were considered congenic when all 96 microsatellite markers were homozygous to C57BL/6j. The deletion of exons 2 and 3 was confirmed by PCR, as reported previously (*21*). Sequences for the wt-specific and mT2-specific primer pairs are identical to those previously reported (*21*). Mice were housed 5 mice per cage, under a 12-hour light/dark cycle with access to Purina chow and water *ad libitum.* All animal procedures reported in this study were performed by CCR staff. All staff and protocols were approved by the NCI Animal Care and Use Committee (ACUC, ASP # LP-003-4) and followed federal regulatory requirements and standards. All components of the intramural NIH ACU program are accredited by AAALAC International procedures were approved.

Cell culture: Lewis lung carcinoma cells stably expressing the Luc2 Luciferase gene (LL/2-Luc2) were obtained from the American Type Culture Collection (ATCC) and cultured in Dulbecco's Modified Eagle Medium (DMEM), 10% fetal bovine serum (FBS), 1% GlutaMAX, 1% penicillin-streptomycin (P-S). LL/2 spheroids were generated at 5000 cells per well using ultra-low attachment round-bottomed 96-well plates (Corning) and cultured for 6 days to allow efficient spheroid formation. Primary murine lung fibroblasts were cultured in Eagle's Minimum Essential Medium, 10% FBS, 1X non-essential amino acids (ThermoFisher), and 1% P-S. An automated cells counter, LUNA-FL (Logos Biosystems Inc.), was used to determine the viability, and cell cultures with greater than 95% viability were used for experiments.

***Preparation of recombinant* TIMP2:** rTIMP2 with a C-terminal 6x-Histidine tag was produced as previously described (*50*). Lyophilized rTIMP2 was resuspended in sterile Hank's balanced salt solution (HBSS) at a concentration of 6ug/100uL and stored at -80° C in single-use aliquots ready for intraperitoneal injection.

Lung fibroblast isolation: Murine lung tissues were minced using two scalpels, then transferred to a 30mL Erlenmeyer flask with 10mL DMEM-F12 media supplemented with 10mM HEPES, 200U/mL collagenase D, 2.4U/mL Dispase, 100U/mL DNA I, and 1% penicillin-streptomycin (P-S). The digestion mix was incubated at 37°C, stirring slowly, for approximately 40 minutes

bioRxiv preprint doi: https://doi.org/10.1101/2023.12.29.573636; this version posted December 29, 2023. The copyright holder for this preprint (which was not certified by peer review) is the author/funder. This article is a US Government work. It is not subject to copyright under 17 USC 105 and is also made available for use under a CC0 license.

(when lung fragments changed color from red to white, forming sticky fibers). 40mL DMEM-F12 10% FBS 1% P-S (DMEM-F12 FM) was added to the digestion mix and mixed extensively by pipetting to resuspend the fragments. The tissue fragments were washed 3x with 20mL DMEM-F12 FM, pelleting the tissue at 500G for 5 minutes in between washes. Tissue fragments were resuspended in 10mL DMEM-F12 FM and plated in 10cm dishes. Stromal cells (fibroblasts) exit the tissue fragments over the next few days, attach to the tissue culture plastic, and begin to proliferate. Between days 7-14, the tissue fragments were washed away, and culture media was changed to EMEM 10% FBS, 1X non-essential amino acids, and 1% P-S to support fibroblast propagation.

Immunoblot analysis: Tissue homogenates containing TCEP were boiled for 3 minutes and loaded into precast 4-20% polyacrylamide gels (Bio-Rad). SDS-PAGE was performed, and proteins were transferred to nitrocellulose membranes using a Trans-Blot Turbo system (Bio-Rad). The membranes were blocked for 1 hour in 2.5% milk TBS 0.1% Tween 20, before immunostaining. The antibodies used are summarized in Supplementary Table 1. Either alpha-tubulin or total protein (Bio-Rad stain-free gels or Ponceau stain) was used to normalize sample loading.

Immunohistochemistry & Immunofluorescence: Immunohistochemistry (IHC) and immunofluorescence (IF) were performed on formalin-fixed paraffin-embedded (FFPE) or frozen tissue embedded in optimal-cutting temperature (OCT) medium using standard laboratory practices for processing, embedding, sectioning, and staining. Primary antibodies are listed in Table S3.

Supplementary Material

Figure S1. (A) Experimental design in a female-only study comparing wt vs mT2 orthotopic tumor growth. (B) Metrics describing the differences in orthotopic LL/2-Luc2 tumor growth between wt and mT2 tumor-bearing female mice. (C) The gating strategy utilized to compare myeloid cell populations in wt and mT2 mice. (D) Design of a pilot orthotopic tumor study investigating the effective dose range for rTIMP2, and (E) in vivo imaging depicting differences in tumor volume across the treatment regimens. (F) Kaplan-Meier survival analysis comparing untreated, 100ug/kg/day, and 200ug/kg/day rTIMP2 treatment regimens in wt mice.

bioRxiv preprint doi: https://doi.org/10.1101/2023.12.29.573636; this version posted December 29, 2023. The copyright holder for this preprint (which was not certified by peer review) is the author/funder. This article is a US Government work. It is not subject to copyright under 17 USC 105 and is also made available for use under a CC0 license.

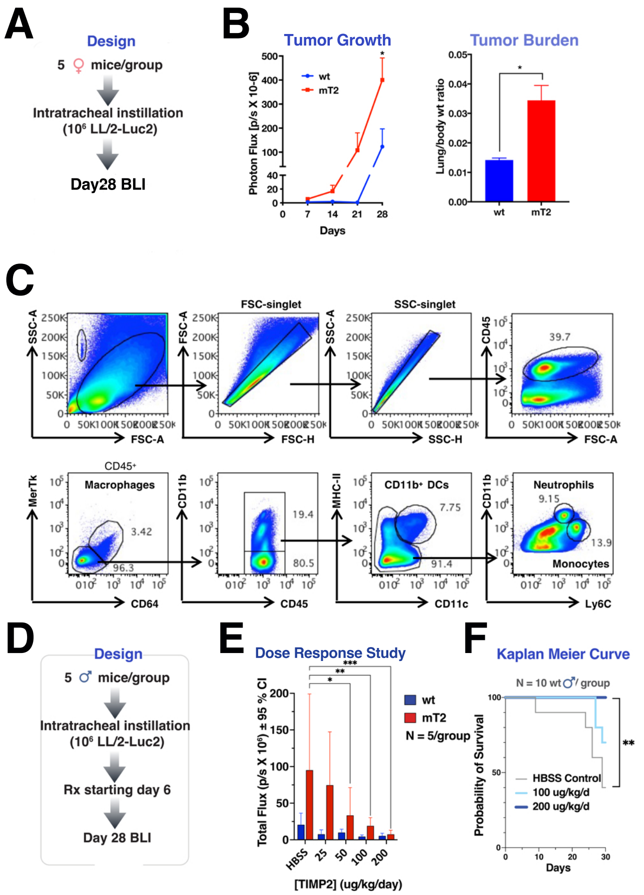

Figure S2. (A) Experimental design in study to examine time frame of lung metastasis from dorsal flank tumors in wt mice. (B) Lung weights (correlating with metastatic burden) of wt mice during the time frame to metastasis outlined in panel A. Data shows that the period between day 7 and day 21 post-tumor inoculation is critical for metastasis formation and development of the metastatic niche.

bioRxiv preprint doi: https://doi.org/10.1101/2023.12.29.573636; this version posted December 29, 2023. The copyright holder for this preprint (which was not certified by peer review) is the author/funder. This article is a US Government work. It is not subject to copyright under 17 USC 105 and is also made available for use under a CC0 license.

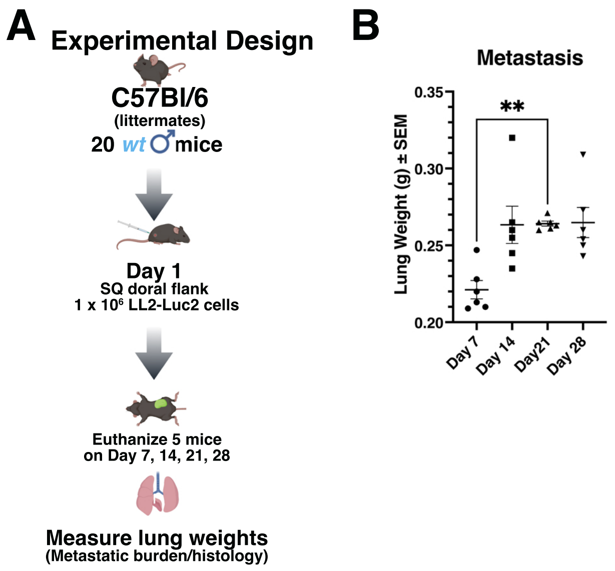

Table S1. Summary of orthotopic Lewis Lung Carcinoma RNA sequencing data and gene set analysis using Ingenuity Pathway Analysis.

Table S2. Summary of subcutaneous heterotopic Lewis Lung Carcinoma RNA sequencing data and gene set analysis using Ingenuity Pathway Analysis.

Table S3. Antibodies and reagent list.

ARRIVE Author Checklist
